# Supplementary material for: Leptospirosis-associated meningitis in an urban tropical endemic setting in northeastern Brazil: Three New cases and a meta-summary of 176 reported cases
Source: PLoS Negl Trop Dis. 2026 May 11;20(5):e0014315. doi: 10.1371/journal.pntd.0014315 (PMC13178981; doi:10.1371/journal.pntd.0014315)
Supplement: Attachment — Submitted filename: PNTD-D-26-00600_reviewer (1) RG.pdf [file pntd.0014315.s001.pdf]

PLOS Neglected Tropical Diseases

Leptospirosis-Associated Meningitis in an Urban Tropical Endemic Setting in Northeastern Brazil: Three New Cases and a Meta-summary of 176 Reported Cases

--Manuscript Draft--

**Manuscript Number:**	PNTD-D-26-00600
**Full Title:**	Leptospirosis-Associated Meningitis in an Urban Tropical Endemic Setting in Northeastern Brazil: Three New Cases and a Meta-summary of 176 Reported Cases
**Short Title:**	Leptospirosis-Associated Meningitis in Northeastern Brazil
**Article Type:**	Research Article
**Keywords:**	Meningitis; Leptospirosis; Zoonosis
**Abstract:**	Background<br><br>Leptospirosis is a globally prevalent zoonotic disease with a wide clinical spectrum of clinical manifestations. Central nervous system involvement is a rare and under-recognized presentation of this infection. Most of the available data on leptospirosis-associated meningitis (LAM) are limited to case reports and small case series, and, to the best of our knowledge, no meta-summary of published case reports focusing specifically on meningeal or encephalic involvement has been conducted to date.<br><br>Methods<br><br>We described a retrospective review of patients with meningitis, encephalitis, or meningoencephalitis admitted to a tertiary infectious diseases referral center in northeastern Brazil. Three cases of LAM were identified in patients with aseptic meningitis. In parallel, a comprehensive literature review was performed, and a meta-summary of reported cases was created. Available CSF parameters were extracted and analyzed using descriptive statistics and boxplot visualization.<br><br>Results<br><br>A total of 809 patients with confirmed meningitis or encephalitis were identified, of whom 447 had aseptic meningitis. Three patients (0.67% of aseptic meningitis cases) were diagnosed with LAM. Clinical presentations included isolated meningitis and meningoencephalitis. In our cohort, CSF analysis showed mild to moderate pleocytosis with lymphocytic predominance, normal to elevated glucose levels, and elevated protein concentrations. Boxplot analysis combining literature data and the three reported cases demonstrated a predominantly lymphocytic CSF profile, mildly increased cellularity, preserved glucose levels, and elevated protein levels, although substantial heterogeneity was observed.<br><br>Conclusion<br><br>Globally, LAM represents an uncommon yet clinically significant cause of aseptic meningitis and meningoencephalitis, particularly in endemic regions. The typical cerebrospinal fluid (CSF) profile demonstrates lymphocytic pleocytosis, mildly to moderately elevated protein levels, and preserved glucose concentrations. Leptospirosis may be associated with a broad spectrum of neurological manifestations, including isolated meningitis, meningoencephalitis, acute encephalitis, and both central and peripheral nervous system involvement. Heightened clinical awareness, along with well-designed prospective studies, is essential to better delineate the full clinical spectrum, underlying pathophysiological mechanisms, and optimal management strategies of LAM.
**Additional Information:**	
**Question**	**Response**
**Government Employee**	No - No authors are employees of the U.S. government.

Powered by Editorial Manager® and ProduXion Manager® from Aries Systems Corporation

Are you or any of the contributing authors an employee of the United States government?<br><br>Manuscripts authored by one or more US Government employees are not copyrighted, but are licensed under a CC0 Public Domain Dedication, which allows unlimited distribution and reuse of the article for any lawful purpose. This is a legal requirement for US Government employees.<br><br>This will be typeset if the manuscript is accepted for publication.	
**Financial Disclosure**<br><br>Enter a financial disclosure statement that describes the sources of funding for the work included in this submission. Review the submission guidelines for detailed requirements. View published research articles from *PLOS Neglected Tropical Diseases* for specific examples.<br><br>This statement is required for submission and **will appear in the published article** if the submission is accepted. Please make sure it is accurate.<br><br>**Funded studies**<br>Enter a statement with the following details:<br>• Initials of the authors who received each award<br>• Grant numbers awarded to each author<br>• The full name of each funder<br>• URL of each funder website<br>• Did the sponsors or funders play any role in the study design, data collection and analysis, decision to publish, or preparation of the manuscript?<br><br>Did you receive funding for this work?	The author(s) received no specific funding for this work.
**Competing Interests**	None.

On behalf of all authors, disclose any competing interests that could be perceived to bias this work.<br><br>This statement will be typeset if the manuscript is accepted for publication.<br><br>Review the instructions link below and PLOS NTDs' competing interests policy to determine what information must be disclosed at submission.	
**Data Availability**<br><br>Provide a **Data Availability Statement** in the box below. This statement should detail where the data used in this submission can be accessed. This statement will be typeset if the manuscript is accepted for publication.<br><br>Before publication, authors are required to make all data underlying their findings fully available, without restriction. Review our PLOS Data Policy page for detailed information on this policy. Instructions for writing your Data Availability statement can be accessed via the Instructions link below.	Data is available upon request.

Manuscript
Click here to access/download;Manuscript;main manuscript leptospirosis associated meningitis.docx

Original Article

Leptospirosis-Associated Meningitis in an Urban Tropical Endemic Setting in Northeastern Brazil: Three New Cases and a Meta-summary of 176 Reported Cases[J]

Short title: Leptospirosis-Associated Meningitis Cases

Authors: Luís Arthur Brasil Gadelha Farias (https://orcid.org/0000-0002-8978-9903)[1,2,3*], Osvaldo Mariano Viana Neto (https://orcid.org/0009-0005-1261-5218)[4], Ednaldo Pereira Lima Sobrinho (https://orcid.org/0009-0008-9048-5561)[4], Caroline Lucena de Almeida Vale (https://orcid.org/0009-0008-6225-2891), Geraldo Bezerra Silva Júnior (https://orcid.org/0000-0002-8971-0994), Elizabeth de Francesco Daher (https://orcid.org/0000-0003-4189-1738), Glaura Fernandes Teixeira de Alcântara (https://orcid.org/0000-0002-4106-2510)[2], Antônio Silva Lima Neto (https://orcid.org/0000-0003-2798-6730), Tania Mara Silva Coelho, Maura Salaroli de Oliveira, Lauro Vieira Perdigão Neto (https://orcid.org/0000-0002-7681-8090)[1,4]

Affiliations

1. Department of Infectious Diseases of Hospital das Clínicas and Laboratório de Investigação Médica – LIM 49, University of São Paulo, São Paulo, Brazil.

2. São José Hospital for Infectious Diseases, Fortaleza, Brazil.

3. Christus University Center (Unichristus), Fortaleza, Brazil.

4. Department of Community Health, Faculty of Medicine, Federal University of Ceará, Fortaleza, Brazil.

5. Health Department of the State of Ceará (SESA-CE), Fortaleza, Brazil.

*Corresponding author: Luís Arthur Brasil Gadelha Farias. Hospital São José de Doenças Infecciosas (HSJ). Rua Nestor Barbosa, 315, Postal Code 60455-610, Fortaleza, Ceará, Brazil. E-mail: luisarthurbrasilk@hotmail.com

Declarations

Ethics approval and consent to participate

This study is part of a larger cohort that was approved by the Research Ethics Committee of the Hospital São José de Doenças Infecciosas (HSJ) (Nº CAAE 52811521.7.0000.5044).

Availability of data and materials

All data supporting the findings of this study are available upon request.

Competing interests

No conflicts of interest were declared by the authors.

Funding statement

No funding was received for this study.

Authors' contributions

LABGF, OMVN: Conception and design of the study. LABGF, OMVN, EPLS: Data acquisition; drafting of the manuscript. GBSJ, EDFD, SFC, LVPN: Methodology and analysis; critical revision of the manuscript; Supervision. GBSJ, EDFD, SFC, LVPN: Critical revision of the manuscript; Supervision and approved the final draft. LVPN: Final approval of the submitted manuscript.

Acknowledgments

We would like to acknowledge Christus University Center and São José Hospital for Infectious Diseases for their valuable support during the research and data collection. We also thank Programa Institucional de Bolsas de Iniciação Científica (PIBIC) at the Federal University of Ceará (UFC) for providing research scholarships to the second and third authors during the development of this study.

Abstract

Background:

Leptospirosis is a globally prevalent zoonotic disease with a wide clinical spectrum of clinical manifestations. Central nervous system involvement is a rare and under-recognized presentation of this infection. Most of the available data on leptospirosis-associated meningitis (LAM) are limited to case reports and small case series, and, to the best of our knowledge, no meta-summary of published case reports focusing specifically on meningeal or encephalic involvement has been conducted to date.

Methods:

We described a retrospective review of patients with meningitis, encephalitis, or meningoencephalitis admitted to a tertiary infectious diseases referral center in northeastern Brazil. Three cases of LAM were identified in patients with aseptic meningitis. In parallel, a comprehensive literature review was performed, and a meta-summary of reported cases was created. Available CSF parameters were extracted and analyzed using descriptive statistics and boxplot visualization.

Results:

A total of 809 patients with confirmed meningitis or encephalitis were identified, of whom 447 had aseptic meningitis. Three patients (0.67% of aseptic meningitis cases) were diagnosed with LAM. Clinical presentations included isolated meningitis and meningoencephalitis. In our cohort, CSF analysis showed mild to moderate pleocytosis with lymphocytic predominance, normal to elevated glucose levels, and elevated protein concentrations. Boxplot analysis combining literature data and the three reported cases demonstrated a predominantly lymphocytic CSF profile, mildly increased cellularity, preserved glucose levels, and elevated protein levels, although substantial heterogeneity was observed.

Conclusion:

Globally, LAM represents an uncommon yet clinically significant cause of aseptic meningitis and meningoencephalitis, particularly in endemic regions. The typical cerebrospinal fluid (CSF) profile demonstrates lymphocytic pleocytosis, mildly to moderately elevated protein levels, and preserved glucose concentrations. Leptospirosis may be associated with a broad spectrum of neurological manifestations, including isolated meningitis, meningoencephalitis, acute encephalitis, and both central and peripheral nervous system involvement. Heightened clinical awareness, along with well-designed prospective studies, is essential to better delineate the full clinical spectrum, underlying pathophysiological mechanisms, and optimal management strategies of LAM.

Key-words: Meningitis; Leptospirosis; Zoonosis;

INTRODUCTION

Leptospirosis is a globally prevalent zoonotic disease caused by *Leptospira* spp., Gram-negative spirochetes that typically use mammals—mainly rodents—as their natural reservoirs[1]. In tropical continental countries such as Brazil, leptospirosis can exhibit high incidence rates, particularly in endemic areas during the rainy season and following flooding events. In the Northeast region, between 2019 and 2022, 2,199 cases of leptospirosis were reported, with a case fatality rate of 13.82%[2]. The bacteria colonize the renal tubules of reservoir hosts and are excreted in their urine, contaminating the environment and enabling transmission to humans, who are considered incidental hosts[1]. The incubation period in humans is variable, ranging from one day to four weeks after exposure, and in survivors, the infection may persist for several months[3].

After contamination, the spirochetes disseminate through the bloodstream during an approximately eight-day period known as the leptospiremic phase, in which a higher pathogen load may result in more severe disease[1]. The subsequent immune phase corresponds to the immunological response to infection[4]. Clinically, leptospirosis can range from a mild febrile illness to a severe icteric–hemorrhagic syndrome with multiple organ dysfunction[5], first described by Adolph Weil in 1886[6]. Despite extensive research, important gaps remain in understanding the pathogenesis of this tropical disease, particularly regarding the extent to which clinical manifestations result from direct bacterial invasion versus host immune-mediated injury[1].

Although it is described and frequently cited as part of the disease spectrum[7], leptospirosis-associated meningoencephalitis remains poorly characterized in the medical literature. Most publications addressing this topic were released before the 21st century, often in low-impact scientific journals, and many of these reports are not available online, hindering the consolidation of current knowledge on leptospirosis-associated meningitis (LAM). Several factors may contribute to this gap: (i) LAM can clinically mimic other causes of aseptic meningoencephalitis, such as viral, mycobacterial or rickettsial infections[8]; (ii) appropriate diagnostic tools for central nervous system involvement are often unavailable in regions where leptospirosis is endemic; and (iii) the empirical use of antibiotics during the course of meningoencephalitis may reduce the likelihood of isolating the pathogen[9].

Consequently, establishing a consistent clinical–epidemiological–laboratory profile of LAM remains challenging, as it may encompass a wide range of neurological syndromes, including meningitis[10], meningoencephalitis[9], acute encephalitis[11], demyelinating diseases[12,13], cerebrovascular disorders[14], focal CNS lesions[15], peripheral neuropathies[16], and Guillain–Barré syndrome[17], among others. Furthermore, several inconsistencies persist across current studies—for instance, some reports describe leptospiral meningitis as an exclusive manifestation of anicteric forms, whereas others demonstrate its occurrence in cases of Weil's disease[18, 19].

Herein, we aim to describe cases of LAM among patients with aseptic meningitis admitted to a tertiary infectious disease center in Ceará, Northeastern Brazil, and to describe their clinical

characteristics. In addition, we conducted an extensive literature review with a meta-summary of case reports, case series, and cohort studies to improve the understanding of the clinical, microbiological, and cerebrospinal fluid profiles of leptospiral meningitis.

METHODS

Asseptic meningitis cohort and leptospiral-associated meningitis cases

This manuscript comprises a retrospective observational study incorporating a case series of leptospirosis-associated meningitis, complemented by a structured literature review and a meta-summary of published cases.

This study comprised a comprehensive review of medical records from patients managed at a statewide referral center for infectious diseases, which receives virtually all cases of meningitis and encephalitis in Ceará, a state in the Northeast region of Brazil with a population of approximately nine million inhabitants.

Definitions

Acute encephalitis syndrome and meningitis were defined according to the 2003 WHO guidelines[20]. Acute encephalitis syndrome was characterized by sudden onset of fever with altered mental status (such as confusion, disorientation, coma, or aphasia) and recent seizures (excluding simple febrile seizures). Meningitis was defined as sudden fever (>38.5 °C rectal or 38.0 °C axillary) accompanied by neck stiffness, altered consciousness, or other meningeal signs. Patients meeting criteria for both conditions were classified as meningoencephalitis[8, 20].

Aseptic meningitis/encephalitis/meningoencephalitis are commonly defined as a syndrome characterized by acute onset of signs and symptoms of meningeal inflammation, cerebrospinal fluid (CSF) pleocytosis and the absence of microorganisms on Gram stain and/or on routine culture[21]. LAM were defined as confirmation of leptospirosis on seric/liquoric serology, microagglutination test, PCR or culture, with the presence of compatible meningeal/encephalical syndrome[9, 22,].

Literature review

A narrative review of the literature was conducted to identify cases of meningitis associated with leptospirosis. Searches were performed in electronic databases, including PubMed, Scopus, and Web of Science, using the terms “meningitis” AND “leptospirosis.” Articles were selected based on online availability and included case reports, reviews, clinical, laboratory, and pathology studies. Only studies published in English were considered, prioritizing those providing detailed information on the neurological presentation of the disease.

Studies describing cases of leptospirosis meningeal syndromes that met the predefined criteria were grouped, as shown in Table 2. Those providing defined CSF parameters were compiled into a separate dataset, from which the following variables were extracted whenever available: cell count (cells/mm³), lymphocytes (%), neutrophils (%), CSF protein (mg/dL), and CSF

glucose (mg/dL). When a study did not report a specific parameter, or when values were presented in non-compatible units (e.g., blood/CSF ratios), that parameter was excluded from the analysis for that study.

For each variable, the corresponding boxplot statistics were obtained (Q1, median [Q2], Q3, lower whisker, and upper whisker) and plotted together with the values from the three cases reported in the present study using Python (Matplotlib/Seaborn). To ensure uniformity and avoid distortion of the final boxplot distributions, extreme cerebrospinal fluid values were excluded according to predefined biological plausibility thresholds. Cases with cellularity above 1300 cells/mm³ or protein concentrations exceeding 500 mg/dL were removed, as such values represented outliers far beyond the expected range for LAM and could disproportionately influence the visualization of central tendency and dispersion. Additionally, entries lacking all CSF quantitative variables were omitted to prevent incomplete cases from biasing the graphical analysis.

Missing data were visualized using a Seaborn heatmap. To ensure that the heatmap reflected the contribution of each study proportionally, the dataset was expanded so that each row represented an individual patient; for series-based studies, each record was replicated according to the reported number of patients.

Results

Aseptic meningitis cohort

Initially, 809 patients with confirmed meningitis/encephalitis/meningoencephalitis were identified. Among them, there were 447 patients with aseptic meningitis. From them, those with negative cultures and RT-PCR FilmArray® meningitis/encephalitis (ME) panel (BioFire Diagnostics, LLC, Salt Lake City, UT, USA), and a clinical suggestive picture underwent *Leptospira* serology. All patients with positive *Leptospira* IgM ELISA and confirmed meningeal syndrome recorded in their medical records were included in our analysis. Of the 447 patients, three patients (0.67%) confirmed leptospiral infection. Laboratory and medical data, including those at admission, were available for the two patients included in the analysis.

Cases description

Case 1. A 22-year-old Brazilian man who works in an urban area as a motorcycle delivery worker, and without previous comorbidities, arrived at the emergency room (ER) with a 7-day history of high fever (Temperature 40ºC), headache, nuchal stiffness, polyarthralgia, macroscopic hematuria and nausea. The patient reported that, one week prior to hospital admission, he experienced prolonged (>1 hour) dermal exposure of the feet to stagnant rainwater on urban streets in the course of his occupational activities. He denied other water-exposure activities and recent travel. Vital signs included a heart rate of 94 bpm, a respiratory rate of 20 rpm, and a peripheral oxygen saturation (SpO_2) of 99% without supplementary oxygen. On physical examination, he presented with mild jaundice and a pain-related facial expression. Neurological examination revealed positive Kernig's and Brudzinski's signs, with

a negative Lasègue sign. The patient was alert, communicative, and fully oriented, with a Glasgow Coma Scale score of 15. Initial laboratory tests revealed elevated bilirubin levels, renal dysfunction, and thrombocytopenia (Table 1). Initial serological tests for anti-HIV 1 and 2 (Fourth-generation antibody/antigen test), hepatitis B and C, hepatitis A IgM ELISA, cytomegalovirus IgM/IgG ELISA, and the Venereal Disease Research Laboratory (VDRL) test were all negative. Cranial computed tomography (CT) was unremarkable. CSF analysis demonstrated findings consistent with aseptic meningitis (Table 2). Based on the clinical presentation, laboratory findings, and epidemiological history - particularly occupational exposure during the rainy season - an anti-Leptospira IgM ELISA (Panbio®, Abbott, Australia) was performed in the serum, yielding a positive result. Microscopic agglutination testing (MAT) was subsequently conducted, demonstrating a titer of 1:400. The serovar was not identified.

The patient was managed initially as bacterial meningitis with intravenous ceftriaxone (2 g, twice daily), vancomycin (15 mg/kg three times daily), and intravenous dexamethasone (12 mg, three times daily). Due to the positive anti-leptospira serology, and CSF findings, vancomycin and dexamethasone were suspended. During hospitalization, the patient experienced resolution of jaundice and improvement of meningeal signs; however, nausea and hyperemesis persisted. An abdominal ultrasound revealed no evidence of biliary lithiasis. Endoscopic ultrasound confirmed the absence of biliary stones. Non-contrast CT of the abdomen and pelvis revealed a diffusely mildly enlarged pancreas with adjacent fat stranding, findings suggestive of acute pancreatitis. Admission to the intensive care unit and renal replacement therapy were not required. Approximately one week later, the patient's vomiting and nausea resolved completely, and full recovery ensued. After a four-week hospitalization, the patient was clinically stable and discharged home with outpatient follow-up.

Case 2. A 66-year-old Brazilian man, a civil construction worker, arrived at the ER with a two-week history of fever, severe headache, polyarthralgia, disorientation, and sensory loss. His medical history was notable for systemic arterial hypertension (SAH) and alcohol abuse. He had a history of a laceration on his left limb during a flood caused by contact with an unknown object. Vital signs included a heart rate of 124 bpm, a temperature of 37,8ºC, a respiratory rate of 16 rpm, and a peripheral oxygen saturation (SpO_2) of 88% without supplementary oxygen. On physical examination, Glasgow Coma Scale score of 11, with marked sensory loss. Neurological examination revealed nuchal stiffness; however, Brudzinski, Kernig, and Lasègue signs were not assessed. Jaundice and edema were absent. He had an erythematous-bullous lesion at the site of the previous laceration, with warmth and edema. On admission, laboratory tests showed elevated bilirubin, anemia, leukocytosis, mild renal dysfunction, and increased C-reactive protein (Table 1). CSF analysis revealed results consistent with aseptic meningitis (Table 2). CT of the left limb showed preserved skin and subcutaneous tissue, with no abnormalities in the periarticular myotendinous structures. An anti-Leptospira IgM ELISA (Panbio®, Abbott, Australia) returned a positive result, while the microscopic agglutination test (MAT) was unavailable at the time.

He was initially treated with ceftriaxone (2 g every 12 hours) and vancomycin (15 mg/kg every 8 hours). Because of sensory loss, he was admitted to the ICU and required orotracheal

intubation. After 4 days, he was extubated and showed improvement in renal function without the need for hemodialysis. Other complications, such as alveolar hemorrhage, were investigated through thoracic CT and ruled out. After a fourteen-day course of ceftriaxone and vancomycin, the patient showed clinical improvement, remained stable, and was discharged home. He returned for a follow-up clinical evaluation six months later and continues to have no sequelae.

Case 3. A 25-year-old eutrophic transgender woman and waste picker was admitted to the emergency department following a convulsive episode two days prior and a newly diagnosed type 2 diabetes mellitus, under suspicion of tetanus. She reported chronic use of benzodiazepines and smoking one cigarette per day. On presentation, she complained of myalgia, calf pain, and stiffness in the neck and trunk, with a measured temperature of 37.8°C. Cardiac, pulmonary, and dermatological examinations were unremarkable. Her complete blood count was within normal limits.

Given a positive epidemiological history for leptospirosis (work in areas with stagnant rainwater) and a compatible clinical picture, an anti-Leptospira IgM ELISA (Panbio®, Abbott, Australia) was requested and returned positive. She was started on ceftriaxone 2g intravenously. A lumbar puncture was performed (Table 1), revealing lymphocytic meningoencephalitis with low cellularity and protein, consistent with leptospiral infection. CT scans were normal.

The patient was hospitalized due to azotemia, evidenced by a serum creatinine of 3.5 mg/dL, serum urea of 115 mg/dL, secondary metabolic acidosis, and hyperkalemia. The azotemia was attributed to rhabdomyolysis, with creatine phosphokinase peaking at 72,000 U/L. She was managed with intravenous fluid therapy, avoiding dialysis throughout hospitalization. During her stay, she remained fully alert and oriented in time and space, without any further convulsive episodes or other neurological abnormalities. Her baseline creatinine was 2.2 mg/dL, with serum urea of 100 mg/dL, and without hyperkalemia or acidosis. She was discharged with follow-up in a chronic kidney disease outpatient clinic.

Literature Review

In this study, we present three rare cases of a common disease - leptospirosis. Given the uncertainties regarding the possible axial and extra-axial manifestations of the disease, we conducted a literature review with a meta-summary of published case reports. Through this approach, we identified 44 articles describing 179 cases with available clinical and cerebrospinal fluid profiles.

The pathogens identified across the studies included several *Leptospira* serovars, predominantly *L. icterohaemorrhagiae, L. canicola, L. interrogans* (including sejroe and bataviae), and *L. santarosai*, as well as reports of *L. grippotyphosa*, *L. australis, L. borgpetersenii Tarassovi*, *L. pomona*, *L. tropica*, and *L. tsaratsovo,* reflecting a broad etiological diversity among LAM cases.

The retrieved articles were summarized in Table 2. The aggregated data show that, among the studies with available information on jaundice, the majority of patients with leptospiral meningitis/meningoencephalitis were anicteric (59.1%), while 40.9% presented with jaundice. Five studies did not express the prevalence of icteric/anicteric patients in their cohort.

The articles that provided cerebrospinal fluid data were allocated to a spreadsheet; total missing data was 44.1% (figure 1). A total of 179 patients had their CSF analyzed. A lymphocytic inflammatory pattern was described, with a median low-to-moderate cellularity (approximately 197 cells/mm³) and wide variation, including a few very high values (figure 2). The lymphocyte ratio is high (median close to 73%), while neutrophils remain low (median close to 18%). However, among the 42 patients for whom cerebrospinal fluid (CSF) neutrophil data were available, 12 (28.6%) demonstrated a predominance of neutrophils in the CSF. Cerebrospinal fluid glucose tends to be within the normal range (median ~60 mg/dL), and protein shows a slight to moderate elevation (median ~67–90 mg/dL), although with rare very high values. Taken together, the findings reinforce a typical profile of aseptic meningitis, with lymphocytic predominance, absent hypoglycorrhachia, and variable proteinorrhachia.

Two reports were excluded from Figure 2 due to markedly elevated protein levels in the CSF, measuring 875 mg/dL and 2590 mg/dL, respectively [15, 23]. Additionally, two cases exceeded the predefined upper threshold for CSF cellularity, with 7800 cells/mm³ and 5000 cells/mm³, and were therefore also removed from the analysis[23].

DISCUSSION

The present study comprises a retrospective cohort of aseptic meningitis cases managed at a tertiary infectious diseases center in northeastern Brazil, with identification and characterization of confirmed cases of leptospiral meningitis/meningoencephalitis/encephalitis. In our cohort, 3/447 (0.67%) of patients with aseptic meningitis had serological evidence compatible with leptospirosis, including one case of isolated meningitis and two cases of meningoencephalitis, all of which exhibited lymphocytic predominance in the cerebrospinal fluid. In contrast, Romero et al. (2010)[24] reported a 58.97% positivity rate for *Leptospira* spp. using molecular methods in cerebrospinal fluid samples from patients with aseptic meningitis, a proportion substantially higher than that observed in our study. This discrepancy may be explained by methodological differences, as PCR-based techniques tend to demonstrate greater sensitivity[8]. In another study conducted in the same region, IgM ELISA positivity in cerebrospinal fluid was observed in 14.6% of cases of meningitis of indeterminate etiology[25]. In large series of leptospirosis, meningeal involvement has been reported in up to 37% of patients, and cerebrospinal fluid pleocytosis in up to 47%[26]. Therefore, the likelihood that test positivity in the context of meningitis merely reflects incidental exposure to *Leptospira* is considered low[9].

Given the scarcity of detailed descriptions of this condition in the literature, a metasummary of reported cases was conducted to delineate the clinical and cerebrospinal fluid (CSF) profile of patients with meningeal syndromes associated with leptospirosis (Figure 2, Table 2). A total of 179 reports—including case reports, case series, and observational studies—were included,

and their clinical and laboratory data were aggregated for analysis. Patients from the present cohort were also incorporated into this dataset. Overall, a predominantly lymphocytic profile was observed, with mildly increased cellularity, normal to elevated CSF glucose levels, and marked elevation of CSF protein (Figure 2). Nevertheless, substantial heterogeneity in CSF findings was evident, with some cases demonstrating neutrophilic predominance—several of which were confirmed by molecular methods in the CSF[9,18,23,27,28,29,30,31], as well as others showing markedly elevated protein and cell counts[23,29,30,32,33]. These findings underscore the broad spectrum of cerebrospinal fluid abnormalities associated with central nervous system leptospirosis and highlight the diagnostic challenges inherent to this condition.

The pathogenesis of LAM remains poorly understood, largely owing to the frequent underdiagnosis of this condition. Molecular-based prevalence studies conducted in endemic areas generally report higher detection rates, suggesting that central nervous system involvement may be more common than clinically recognized[24]. The frequent use of antibiotics in cases of acute meningitis or systemic leptospirosis contributes substantially to this limitation[23,34]. Nevertheless, experimental studies and clinicopathological reports of central nervous system infection by *Leptospira* spp. provide important insights into the underlying mechanisms.

In milder forms of the disease, it remains unclear to what extent neurological manifestations result from direct pathogen-mediated injury or from host immune responses[1,35]. Similar to the systemic infection, central nervous system involvement is thought to follow a biphasic course[1,35]. There is substantial evidence that, after penetrating host barriers, pathogenic *Leptospira* rapidly disseminate hematogenously, leading to the acute leptospiremic phase[35]. During this stage, the organism can be isolated from multiple tissues, including the central nervous system, often without inducing significant inflammatory responses, which explains the absence of pleocytosis or overt inflammatory changes in some cases[35, 36]. Clinically, this phenomenon is supported by studies demonstrating molecular evidence of *Leptospira* in the central nervous system despite unremarkable inflammatory parameters[23]. In contrast, the immune phase of the disease is believed to account for the majority of neurological manifestations, including meningitis, meningoencephalitis, and encephalitis, driven by antigen–antibody–mediated hypersensitivity reactions[36]. Although uncommon, these cases often demonstrate CSF positivity by serological methods such as MAT and ELISA[24, 37, 38, 39,40,41,42]. Importantly, the detection of *Leptospira* DNA in patients with LAM does not necessarily indicate active infection, as it may reflect residual genetic material rather than viable organisms capable of replication[15].

Counterintuitively, the study by Murgatroyd (1937) described a case of chronic leptospiral meningitis with compatible clinical and CSF findings in which *Leptospira* was isolated from CNS by xenodiagnosis[43]. This observation demonstrates that leptospirosis may present with cerebrospinal fluid pleocytosis and active central nervous system infection, rather than representing solely an immune-mediated phenomenon[43]. In that case, the patient exhibited immunological anergy to their infecting *Leptospira* strain, requiring experimental administration of equine antileptospiral serum for disease control, suggesting that chronicity

resulted from either low bacterial virulence or impaired host immune response[43]. In a convergent manner, Wilson et al. (2014) reported a case of LAM in an adolescent with a four-month history of meningoencephalitis who had a previous diagnosis of severe combined immunodeficiency[44]. This finding supports the hypothesis that persistence of *Leptospira* infection may occur when pathogen clearance after the bacteremic phase is impaired, whether due to host immune deficiency or intrinsic characteristics of the infecting strain. Thus, isolation of the organism may still occur during the so-called "immune phase."Regardless of complement system integrity, effective immune control of leptospirosis relies on the generation of functional, pathogen-specific antibodies. Host-related factors—including comorbidities, immunosuppression, and genetic polymorphisms—play a critical role in shaping the clinical spectrum of disease[1]. Other studies likewise support the possibility of clinically apparent central nervous system infection during the bacteremic phase of leptospirosis[18, 40].

The immunological nature of LAM is further supported by one study, in which the patient developed meningeal symptoms only after the initiation of antibiotic therapy, consistent with a Jarisch–Herxheimer reaction[31]. This phenomenon, characterized by the release of bacterial cell wall components and subsequent inflammatory cytokine surge, is well described in infections such as leptospirosis and syphilis[45]. Histopathological data remain scarce; however, findings reveal cerebral edema and congestion, along with mixed inflammatory infiltrates composed of histiocytes, lymphocytes, and plasma cells[46]. Microglial nodules were also observed across multiple brain regions. Additional findings included perivascular hemorrhage and demyelination, accompanied by varying degrees of lymphohistiocytic infiltration and vascular leakage, consistent with a post-infectious acute disseminated encephalomyelitis–like process without spinal cord involvement. Other studies corroborate these observations, demonstrating predominantly perivascular lymphohistiocytic infiltration within the periarachnoid space[44].

In line with neuropathological findings, LAM is strongly associated with cerebrovascular involvement. Although the exact mechanisms remain unclear, it is widely accepted that immune-mediated vasculitis and/or direct vascular invasion play a central role, contributing not only to intracranial hemorrhage but also to cranial nerve involvement, both of which are frequently observed in this condition[40,47,48]. Additionally, *Leptospira* spp. can interfere with the fibrinolytic system, coagulation pathways, platelet function, and endothelial activation, thereby increasing the risk of hemorrhagic complications[49]. A population-based cohort study from Taiwan demonstrated an increased risk of hemorrhagic stroke among patients with newly diagnosed leptospirosis, even in the absence of overt meningeal involvement[50]. In the reported cases of LAM, cerebrovascular manifestations may include lacunar infarction[13,46,48], subarachnoid hemorrhage[40,51], territorial ischemic stroke[46], Moyamoya-like vasculopathy[8,14], extra-axial hemorrhage[52], and cerebral venous thrombosis[53], among other presentations.

The spectrum of encephalic involvement in leptospirosis is broad and remains incompletely understood, and a comprehensive review of all associated neurological manifestations is beyond the scope of this work. Molecular studies in LAM have expanded understanding of both diagnostic strategies and disease pathophysiology[8,13,24,27,44,54,55]. Evidence suggests that

cerebrospinal fluid PCR is more sensitive than serological assays, enabling earlier diagnosis and therapeutic intervention[24]. However, its use remains limited in many endemic regions, contributing to the underrecognition of LAM in patients presenting with aseptic meningitis[56].

Treatment of leptospirosis should be initiated based on clinical suspicion, as early antimicrobial therapy is essential regardless of whether neurological involvement results from immune-mediated mechanisms or direct bacterial invasion. This study has several limitations, including the lack of cerebrospinal fluid confirmation of *Leptospira* infection, the scarcity of available literature on LAM—often marked by incomplete or heterogeneous data—and its retrospective design.

An estimated one million cases of leptospirosis occur worldwide each year, accounting for approximately 60,000 deaths[1]. Even in its classic clinical presentations, the disease is frequently underrecognized; atypical manifestations further complicate diagnosis and may contribute to substantial underdiagnosis. Nevertheless, these findings contribute meaningfully to the understanding of central nervous system involvement in this neglected tropical disease. Future prospective studies are needed to further elucidate the pathophysiology and clinical spectrum of LAM.

REFERENCES

1) Rajapakse S, Fernando N, Dreyfus A, Smith C, Rodrigo C. Leptospirosis. Nat Rev Dis Primers. 2025;11(1):32. https://doi.org/10.1038/s41572-025-00614-5

2) Silva MDM, Santos RS, Argolo MF, Silva Neto FD, Santos AA, Lima BF, Almeida ABM, Avila MP, Oliveira RJ, Silva FS, et al. Avaliação da taxa de letalidade devido à leptospirose nos estados do Nordeste brasileiro entre os anos de 2019 a 2022. Braz J Infect Dis. 2023;27(S1):103512. https://www.bjid.org.br/en-avaliacao-da-taxa-de-letalidade-articulo-S1413867023007729

3) Evangelista KV, Coburn J. Leptospira as an emerging pathogen: a review of its biology, pathogenesis and host immune responses. Future Microbiol. 2010; 5(9):1413-25. https://doi.org/10.2217/fmb.10.102

4) Brito T, Silva AMG, Abreu PAE. Pathology and pathogenesis of human leptospirosis: a commented review. Rev Inst Med Trop Sao Paulo. 2018; 60:e23 https://doi.org/10.1590/S1678-9946201860023

5) Rajapakse S. Leptospirosis: clinical aspects. Clin Med (Lond). 2022; 22(1):14–7. https://doi.org/10.7861/clinmed.2021-0784

6) Wellcome Collection. Wellcome Collection – item j9y5q3sw. Available form: https://wellcomecollection.org/works/j9y5q3sw/items?canvas=5. Accessed 27 Dec 2025.

7) Bennett JE, Dolin R, Blaser MJ. Mandell, Douglas, and Bennett's principles and practice of infectious diseases. 9th ed. Philadelphia: Elsevier; 2020.

8) Dittrich S, Rattanavong S, Lee SJ, Panyanivonga P, Craig SB, Tulsiani SM, Blacksell SD, Dance DAB, Dubot-Pérès A, Sengduangphachanh A, et al. Orientia, rickettsia, and leptospira pathogens as causes of CNS infections in Laos: a prospective study. Lancet Glob Health 2015; 3: e104–12. https://doi.org/10.1016/S2214-109X(14)70289-X

9) Jha S, Ansari MK. Leptospirosis presenting as acute meningoencephalitis. J Infect Dev Ctries 2010; 4(3):179-82. https://doi.org/10.3855/jidc.646

10) Fox EG, Fyfe WM. Leptospiral Meningitis: Report of a Case. Glasgow Med J. 1951 Oct;32(10):311–2. https://pubmed.ncbi.nlm.nih.gov/14887958/

11) Puca E, Majko J, Puca E, Qyra E, Gega A, Pipero P. Acute encephalitis as initial presentation of leptospirosis. J Infect Dev Ctries. 2017; 11(4):361-3. https://doi.org/10.3855/jidc.8613

12) Singh P, Gupta A, Saggar K, Kaur M. Acute disseminated encephalomyelitis subsequent to Leptospira infection. Ann Trop Med Public Health. 2011;4(2):133–5. https://doi.org/10.4103/1755-6783.85771

13) Chen S-X, Han D-K, Liu Y, Ye Z-H, Lu K, Xu B, Mai H-q. Leptospira infection complicated by demyelinating disease: A case report. Front. Neurol. 2022; 13:1021364. https://doi.org/10.3389/fneur.2022.1021364

14) Ximin L, Xuzhong R, Zhuan C, Burun Y, Shangpu H, Yunhe G. Moyamoya disease caused by leptospiral cerebral arteritis. Chin Med J. 1980; (93)9: 599-604. https://mednexus.org/doi/epdf/10.5555/cmj.0366-6999.93.09.p599.01

15) Sakellaridis N, Panagopoulos D, Androulis A. Neuroleptospirosis with hydrocephalus and very elevated cerebrospinal fluid protein. South Med J. 2009;102(5):549-50. https://doi.org/10.1097/smj.0b013e3181a0ae80

16) Pradhan S; Tandon R; Kishore J. Combined involvement of muscle, nerve, and myoneural junction following Leptospira infection. Neurol India. 2012;60(5):514–6. https://doi.org/10.4103/0028-3886.103202

17) Gokalp C, Yildiz C, Tunc B, Kurultak I, Ustundag S. Guillain-Barre syndrome variant as a rare complication of leptospirosis. J Travel Med. 2021;28(1):taaa143. https://doi.org/10.1093/jtm/taaa143

18) Hickey, K.M. Leptospiral meningitis. Ir J Med Sci. 1954; 29: 32–6. https://doi.org/10.1007/BF02954380

19) Cargill Jr WH, Beeson pb. The value of spinal fluid examination as a diagnostic procedure in Weil's disease. Ann Intern Med. 1947; 27(3):396-400. https://doi.org/10.7326/0003-4819-27-3-396

20) World Health Organization. WHO guidelines on meningitis diagnosis, treatment and care. Geneva: World Health Organization; 2025. ISBN: 978-92-4-010804-2. Disponível em: https://www.who.int/publications/i/item/9789240108042.

21) Tapiainen T, Prevots R, Izurieta HS, Abramson J, Bilynsky R, Bonhoeffer J, Bonnet MC, Center K, Galama J, Gillard P, et al. Aseptic meningitis: Case definition and guidelines for collection, analysis and presentation of immunization safety data. Vaccine. 2007; 25(31):5793-802. https://doi.org/10.1016/j.vaccine.2007.04.058

22) Nabity SA, Araújo GC. Hagan JE. Damião AO. Reis MG. Ko AI. Ribeiro GS. Anicteric Leptospirosis-Associated Meningitis in a Tropical Urban Environment, Brazil. Emerg Infect Dis. 2020; 26(9):2190–2. https://doi.org/10.3201/eid2609.191001

23) Abdelrahim NA, Fadl-Elmula IM, Hartskeerl RA, Ahmed A, Goris M. Are Pathogenic Leptospira a Possible Cause of Aseptic Meningitis in Suspected Children in Sudan?. Res Rep Trop Med. 2021;12:267-74. https://doi.org/10.2147/rrtm.s339058

24) Romero EC, Blanco RM, Yasuda PH. Aseptic meningitis caused by Leptospira spp diagnosed by polymerase chain reaction. Mem. Inst. Oswaldo Cruz. 2010; 105(8): 988-92. https://doi.org/10.1590/S0074-02762010000800007

25) Silva MV, Camargo ED, Batista L, Vaz AJ, Ferreira AW, Barbosa PR. Application of anti-Leptospira ELISA-IgM for the etiologic elucidation of meningitis. Rev Inst Med Trop Sao Paulo. 1996;38(2):153–6. https://doi.org/10.1590/S0036-46651996000200011

26) Heath CW Jr, Alexander AD, Galton MM. Leptospirosis in the United States: analysis of 483 cases in man, 1949–1961. N Engl J Med. 1965;273(17):915–22. https://doi.org/10.1056/NEJM196510212731706

27) Waggoner JJ, Soda EA, Seibert R, Grant P, Pinsky BA. Molecular detection of Leptospira in two returned travelers: higher bacterial load in cerebrospinal fluid versus serum or plasma. Am J Trop Med Hyg. 2015;93(2):238-40. https://doi.org/10.4269/ajtmh.15-0174

28) Nordholm AC, Omland LH, Villumsen S, Al-Subeihe I, Katzenstein TL. Leptospirosis meningitis transmission from a pet mouse: a case report. J Med Case Reports. 2019;13(1):362. https://doi.org/10.1186/s13256-019-2265-7

29) Samkar AV, Beek DV, Stijnis C, Goris M, Brouwer MC. Suspected leptospiral meningitis in adults: report of four cases and review of the literature. Neth J Med. 2015; 73(10):464-70. https://pubmed.ncbi.nlm.nih.gov/26687262/

30) Alani FS, Mahoney MP, Ormerod LP, Wright PA, Garrues M. Leptospirosis presenting as atypical pneumonia, respiratory failure and pyogenic meningitis. J Infect.1993; 27(3):281-3. https://doi.org/10.1016/0163-4453(93)92155-p

31) Tattevin P, Jauréguiberry S, Michelet C. Meningitis as a possible feature of the Jarisch-Herxheimer reaction in leptospirosis. Eur J Clin Microbiol Infect Dis. 2003; 22(7):449. https://doi.org/10.1007/s10096-003-0957-8.

32) Coffey JH, Dravin I, Dine WC. Swineherd’s disease (aseptic meningitis) due to Leptospira pomona. JAMA. 1951;147(10):949–950. https://doi.org/10.1001/jama.1951.73670270001013

33) Watkins, ED. Meningitis Due to Leptospira Canicola. Medical Memoranda. BMJ. 1951;1(4700):227. https://doi.org/10.1136/bmj.1.4700.227

34) Hubbert WT, Humphrey GL. Epidemiology of Leptospirosis in California: A Cause of Aseptic Meningitis. Calif Med. 1968;108(2):113–7. https://pubmed.ncbi.nlm.nih.gov/5639940/

35) Feigin RD, Anderson DC, Heath CW. Human Leptospirosis. CRC Critical Reviews in Clinical Laboratory Sciences,1975; 5(4), 413–67. https://doi.org/10.3109/10408367509107050

36) Levett PN. Leptospirosis. Clin Microbiol Rev. 2001;14(2):296–326. https://doi.org/10.1128/CMR.14.2.296-326.2001

37) Romero EC, Billerbeck AE, Lando VS, Camargo ED, Souza CC, Yasuda PH. Detection of Leptospira DNA in patients with aseptic meningitis by PCR. J Clin Microbiol. 1998;36(5):1453–5. https://doi.org/10.1128/jcm.36.5.1453-1455.1998

38) Torre D, Giola M, Martegani R, Zeroli C, Fiori GP, Ferrario G, Bonetta G. Aseptic meningitis caused by Leptospira australis. Eur J Clin Microbiol Infect Dis. 1994;13(6):496–7. https://doi.org/10.1007/BF01974640

39) Ross CA, Ives JC. Serological diagnosis in leptospiral aseptic meningitis. Lancet. 1960; 2(7163):1278–9. https://doi.org/10.1016/S0140-6736(60)92467-3

40) Bezerra HM, Ataíde Júnior L, Hinrichsen SL, Travassos FM, Travassos PTC, Silva MJB, Silva MB. Comprometimento do sistema nervoso na leptospirose. I. Avaliação dos aspectos neurológicos. Arq Neuropsiquiatr. 1993;51(4):457–63. https://doi.org/10.1590/S0004-282X1993000400006

41) Bezerra HM, Ataíde Júnior L, Hinrichsen SL, Travassos FM, Travassos PTC, Silva MJB, Silva MB. Comprometimento do sistema nervoso na leptospirose. II. Avaliação do líquido cefalorraqueano. Arq Neuropsiquiatr. 1993;51(4):464–8. https://doi.org/10.1590/S0004-282X1993000400007

42) Bezerra HMC, Ataíde Júnior L, Hinrichsen SL, Travassos FM, Travassos PTC, Silva MJB, Silva MB. Comprometimento do sistema nervoso na leptospirose. III. Reações imunológicas no sangue e líquido cefalorraqueano. Arq Neuropsiquiatr. 1993;51(4):469–74. https://doi.org/10.1590/S0004-282X1993000400008

43) Murgatroyd F. Chronic meningitis in Weil's disease. Br Med J. 1937;1(3965):7–11. https://doi.org/10.1136/bmj.1.3965.7

44) Wilson MR, Naccache SN, Samayoa E, Biagtan M, Bashir H, Yu G, Salamat SM, Somasekar S, Federman S, Miller S, et al. Actionable Diagnosis of Neuroleptospirosis by Next-Generation Sequencing. N Engl J Med. 2014; 370(25):2408–17. https://doi.org/10.1056/nejmoa1401268

45) Friedland JS, Warrell DA. The Jarisch-Herxheimer reaction in leptospirosis: possible pathogenesis and review. Rev Infect Dis.1991;13(2):207-10. https://doi.org/10.1093/clinids/13.2.207

46) Mathew T, Satishchandra P, Mahadevan A, Nagarathna S, Yasha TC, Chandramukhi A, Subbakrishna DK, Shankar SH. Neuroleptospirosis - revisited: experience from a tertiary care neurological centre from south India. Indian J Med Res. 2006;124(2):155-62. Available from: https://pubmed.ncbi.nlm.nih.gov/17015929/

47) Bouazzaoui AE, Houari N, Arika A, Belhoucine I, Boukatta B, Sbai H, Alami NE, Kanjaa N. Facial palsy associated with leptospirosis. Eur Ann Otorhinolaryngol Head Neck Dis .2011; 128(5):275-7. https://doi.org/10.1016/j.anorl.2011.02.011

48) Bismaya K, Dev P, Favas TT, Pathak A. Neuro-Leptospirosis: Experience from a tertiary center of North India. Rev Neurol (Paris). 2023; 179(3):238-43. https://doi.org/10.1016/j.neurol.2022.06.009

49) Vieira ML, Herwald H, Nascimento ALTO. The interplay between host haemostatic systems and Leptospira spp. infections. Crit Rev Microbiol. 2020;46(2):121–135. https://doi.org/10.1080/1040841X.2020.1735299

50) Li KY, Chou MC, Wei JC-C, Lin MC, Hung YM, Chang R. Newly diagnosed leptospirosis and subsequent hemorrhagic stroke: a nationwide population-based cohort study. Stroke. 2021;52(3):913–21. https://doi.org/10.1161/strokeaha.120.029998

51) Herrán de la Gala D, Law-Ye B. Basilar meningitis and hydrocephalus in neuroleptospirosis. Acta Neurol Belg. 2024; 124(2):641–3. https://doi.org/10.1007/s13760-024-02502-3

52) Lessa I, Cortes E. Cerebrovascular accident as a complication of leptospirosis. Lancet.1981; 2(8255):1113. https://doi.org/10.1016/s0140-6736(81)91315-5

53) Turhan V, Senol MG, Sonmez G, Oncul O, Cavuslu S, Tanridag O. Cerebral venous thrombosis as a complication of leptospirosis. J Infect. 2006; 53(6):e247-9. https://doi.org/10.1016/j.jinf.2006.02.013

54) Brown PD, Carrington DG, Gravekamp C, van de Kemp H, Edwards CN, Jones SR, Prussia PR, Garriques S, Terpstra WJ, Levett PN. Direct detection of leptospiral material in human postmortem samples. Res Microbiol. 2003;154(8):581–6. https://doi.org/10.1016/s0923-2508(03)00166-9

55) Merien F, Baranton G, Perolat P. Comparison of polymerase chain reaction with microagglutination test and culture for diagnosis of leptospirosis. J Infect Dis.1995;172(1):281-5. https://doi.org/10.1093/infdis/172.1.281

56) Wang N, Han YH, Sung JY, Lee WS, Ou TY. Atypical leptospirosis: an overlooked cause of aseptic meningitis. BMC Res Notes. 2016;9:154. https://doi.org/10.1186/s13104-016-1964-z

57) Díaz-Rivera RS, Ramos-Morales F, Benenson AF, Hall HE, Marchand EJ. Leptospiral meningitis. Arch Intern Med. 1959;103(6):886–96. https://doi.org/10.1001/archinte.1959.00270060038006

58) Bandara AGNMK, Kalaivarny G, Perera N, Indrakumar J. Aseptic meningitis as the initial presentation of Leptospira borgpetersenii serovar Tarassovi: two case reports and a literature review. BMC Infect Dis. 2021;21(1):488. https://doi.org/10.1186/s12879-021-06200-w

59) de Souza AL, Sztajnbok J, Marques SR, Seguro AC. Leptospirosis-induced meningitis and acute renal failure in a 19-month-old male child. J Med Microbiol. 2006; 55(Pt 6):795–7. https://doi.org/10.1099/jmm.0.46486-0

60) Karande DS. Patil S, Kulkarni M, Joshi A, Bharadwaj R. Acute Aseptic Meningitis as the Only Presenting Feature of Leptospirosis. The Pediatric Infectious Disease Journal. 2005; 24(4):390-1. https://doi.org/10.1097/01.inf.0000157351.44089.7e

61) Gancheva GI, Kostadinova MA, Kostadinova PI. Involvement of central nervous system in leptospirosis. J Biomed Clin Res. 2009;2(2):109–14. Available from: https://www.researchgate.net/publication/321344758_INVOLVEMENT_OF_CENTRAL_NERVOUS_SYSTEM_IN_LEPTOSPIROSIS

62) Panicker JN, Mammachan R, Jayakumar RV. Primary neuroleptospirosis. Postgrad Med J. 2001;77(911):589–590. https://doi.org/10.1136/pmj.77.911.589

63) Middleton JE. Canicola Fever with Neurological Complications. Br Med J. 1955;2(4930):25–6. https://doi.org/10.1136/bmj.2.4930.25

64) Marotto PC, Marotto MS, Santos DL, Souza TN, Seguro AC. Outcome of leptospirosis in children. Am J Trop Med Hyg. 1997;56(3):307-10. https://doi.org/10.4269/ajtmh.1997.56.307

65) Bhatt M, Rastogi N, Soneja M, Biswas A. Uncommon manifestation of leptospirosis: a diagnostic challenge. BMJ Case Rep.2018;2018:bcr2018225281. https://doi.org/10.1136/bcr-2018-225281

66) Bigham RS Jr. Benign aseptic meningitis due to Leptospira grippotyphosa: second report of clinical infection in the United States. AMA Arch Intern Med. 1953;92(4):587–590. https://doi.org/10.1001/archinte.1953.00240220135016

67) Mondal S, Ete T, Sinha D, Sarkar S, Chakraborty A, Nag A, Pal J, Ghosh A. Leptospirosis complicated with meningoencephalitis and pancreatitis: a case report. International Journal of Medical Research & Health Sciences. 2014;3(2):477-9. http://dx.doi.org/10.5958/j.2319-5886.3.2.100

FIGURES AND TABLES

Table 1. Cerebrospinal fluid profile of patients with severe leptospirosis-associated meningitis.

	Cellularity (cells/mm^3)	Differential (Linf/Mono/Neu/Eos) [%]	Glucose (mg/dL)	Protein (mg/dL)	ADA (IU/L)	Cultures and bacterioscopy*	PCR**
Case 1	33	75/10/14/01	76,0	31,9	2,2	-	-
Case 2	18	81/05/14/00	132,0	93,0	4,9	-	-
Case 3	9	85/05/10/00	64,8	54,4	—	-	-

"-": Negative results. *Cultures for mycobacteria, fungi, and pyogenic pathogens. **FilmArray® meningitis/encephalitis (ME) panel (BioFire Diagnostics, LLC, Salt Lake City, UT, USA).

Table 2. Studies and cases on leptospirosis-associated meningitis in medical literature.

Study	Age	Country	Type	Icteric/anicteric LAM's	CSF findings	Diagnosis	Interval between fever and meningeal syndrome	Findings	Serovar/Strain
Nabity et al (2020)[22]		Brazil	Retrospective cohort	0/5	⅘ with monomorphic CSF pattern.	MAT + and clinical LAM diagnosis	-	Lower mean CSF cell count and protein ($p<0.05$). 1,7% prevalence among aseptic meningitis patients.	3 *L. icterohaemorrhagicae*, 2 *L. canicola* (presumed).
Nordholm et al (2019)[28]		Denmark	Case report	0/1	Neutrophilic pattern (56%)	MAT+ and clinical LAM diagnosis	15 days	Report of pet mice leptospirosis.	*Leptospira interrogans* serovar sejroe
Wilson et al (2014)[44]		United States of America	Case report	0/1	Lymphocytic pattern (52%). Elevated ACE in CSF.	CSF NGS	0 days	Chronic neuroleptospirosis in a patient with adenosine deaminase deficiency. MRI with hyperintensities in basal ganglia + leptomeningitis.	*L. santarosai*
Wang et al (2016)[56]		Taiwan	Case report	0/1	Lymphocytic pattern (91%), 24cmH_2O of opening pressure	MAT + clinical LAM diagnosis	2 days	Absence of kidney injury, disease	*L. interrogans* serovar bataviae and *L. santarosai* serovar Shermanii
Díaz-Rivera et al (1958)[57] - icteric		Puerto Rico	Prospective cohort	9/0	Lymphocytic pattern (mean value: 85,7%)	Serology	8,1 days (mean)	11,1% of cases with leptospiral CSF isolation. Three punctures with yellow collor.	*L. icterohaemorrhagiae*
Díaz-Rivera et al (1958)[57] - anicteric		Puerto Rico	Prospective cohort	0/7	Lymphocytic pattern (mean value: 62,0%)	Serology	9,3 days (mean)	14,3% of leptospiral CSF isolation.	*L. icterohaemorrhagiae*

Bismaya et al (2022)[48]	India	Retrospective cohort	0/7	Lymphocytic pattern in 100% of patients	Serology and PCR	-	14,0cmH_2O of mean opening pressure. 2 with abnormal MRI.	*L. interrogans* (probable)
Gala et al[51] (2024)	Belgic	Case report	1/0	Lymphocytic pattern.	PCR	9 days	MRI exhibiting subarachnoid exsudates, with smooth leptomeningeal enhancement in and communicating hydrocephalus. Xanthochromic PCR. Patient died.	-
Samkar et al[29] (2015)	Netherlands	Case series	0/1	2 Lymphocytic, 2 neutrophilic (mean: 80,5%) pattern	MAT/Serology/PCR/Culture	-	In the review, a significantly increased mortality were found in patients not treated with antibiotics, when compared to those treated (13% vs 2%; p = 0.04).	Presumed serogroups: Grippotyphosa, Australis, and Mini.
Bandara et al[58] (2021)	Sri Lanka	Case series	0/2	1 Lymphocytic, 1 neutrophilic pattern	MAT	7,5 days (mean)	All alive.	*L. borgpetersenii* serovar Tarassovi
Souza et al[59] (2006)	Brazil	Case report	0/1	Lymphocytic pattern (59%)	MAT		Pediatric case. Normal CT scan. Long-term evolution favourable.	*L. interrogans* serovar icterohaemorrhagiae
Mathew et al[46] (2006)	India	Mixed cohort	-	Lymphocytic pleocytosis in 72%.	MAT		Normal CT scan on 18/27, 7/27 with diffuse cerebral oedema. 5/22 of CSF MAT samples were positive. 26% died. The two	-

							significant parameters for mortality were elevated CSF protein and the degree of altered sensorium at admission. Higher protein level in patients who died (p<0.001)	
Alani et al[30] (1993)	United Kingdom	Case report	1/0	Neutrophilic pleocytosis (95%).	Serology/MAT	-	-	--
Karande et al[60] (2005)	India	Case report	0/1	Lymphocytic pleocytosis (94%).	Serology/MAT	5 days	CT scan revealed inflammation along the tentorium, the ambient cisterns and the left sylvian fissure.	*L. interrogans* serovar icterohaemorrhagiae
Tattevin et al[31] (2003)	France	Case report	0/1	Neutrophilic pleocytosis (90%).	MAT	-	The meningeal syndrome was started four hours after the administration of amoxicillin, and disappeared two days later.	*L. grippotyphosa.*
Jha et al[9] (2010)	India	Case Report	1/0	Lymphocytic pleocytosis (90%).	Serology	-	Normal cranial imaging.	-
Hickey et al[18] (1954)	Ireland	Case series	1/4	3 cases with lymphocytic and one with neutrophilic pleocytosis.	MAT	-	2 cases with concomitant herpes labialis. One patient with meningitis presented with negative MAT during first symptom week, with posterior positivation.	-
Abdelghani et al[23] (2021)	Sudan	Prospective cohort	0/9	> 70% with neutrophil predominance.	PCR	-	Use of PCR methods in children of the emergency department with	-

								pyogenic meningitis diagnosis. 77.8% aged <1 year. Normal CSF in two patients, one with *S. pneumoniae* coinfection, and two with HHV6 coinfection. Average of 2.5 days between onset and lumbar puncture.	
Romero et al[24] (2010)	29,5 (mean)	Brazil	Prospective cohort	0/29	-	CSF PCR/MAT	-	Without laboratorial data. 29 CSF PCR+, 2 CSF MAT+/PCR-, 5 CSF PCR+/MAT+. 0 positive CSF cultures.	-
Romero et al[37] (1998)	-	Brazil	Prospective cohort	0/41	-	CSF PCR/MAT	-	Without laboratorial data. 39.80% CSF PCR+, 3.88 CSF MAT+, 8.74% CSF ELISA+/MAT+.	-
Torre et al[38] (1994)		Italy	Case report	0/1		CSF MAT	-	Normal CT contrast scan.	*L. australis* serovar bratislava (strain Riccio 66).
Silva et al[25] (1996)	Rangingfrom 5 to 15 years	Brazil	Prospective cohort	0/25	64% with neutrophil predominance	CSF Serology	-	-	-
Ross et al[39] (1960)	-	Scotland	Prospective cohort	0/13	-	CSF serology/complement-fixation	-	13/250 tested patients with both serology and complement-fixation positive.	-

Bezerra et al[40,41,42] (1993)	-	Brazil	Prospective cohort	59/18		CSF/Seric MAT		Normality of physical neurological exam in 89,61%. Meningeal syndrome more common in icteric patients (67,9% *vs.* 32,1%). Abnormal CSF more common in icteric patients (81,3% *vs.* 18,8%). Meningoencephalitis/polyneuritis more common in icteric patients. CSF abnormalities in 95.52%, 88,01% with lymphomononuclear predominance. 74,62% with hyperproteinorrhachia. Positive serology of CSF in 64,18%. Higher CSF glycemic levels.	*L. icterohaemorragiae* (66,23%), *L. canicola* (5,19%).
Gancheva[61] et al (2009)	-	Brazil	Case-control study	-		MAT/Serology (?)		20/94 cases with CNS involvement. 16 with meningitis, 4 with meningoencephalitis. 75% with characteristic neurological examination. Intact cranial nerves. Coma in 20%, seizures in 15%.	*L. tropica* and *L. tsaratsovo* as the most prevalent
Panicker et al[62] (2001)	-	India	Retrospective cohort	-	Predominant lymphomononuclear	Serology	-	Prevalence of various neurological syndromes, but aseptic meningitis/meningoencephalitis predominated (16/40).	-
Coffey et al[32] (1951)	27	United States of America	Case report	0/1	Lymphocytic pleocytosis (86%)	MAT	-	-	*L. pomona*

Watkins[33] (1951)	32	England	Case report	0/1	Lymphocytic pleocytosis (73%)	Serology	0 days	Evidence of infection in the patient's dog	*L. canicola* and *L. icterohaemorrhagiae* (concomitantly)
Murgatroyt[43] (1937)	35	England	Case report	1/0	Neutrophilic pleocytosis (60%).	Xenodiagnosis and MAT	42 days	The patient had chronic meningitis before an almost complete clinical resolution of Weil's disease. The dark ground illumination of CSF was negative and the diagnosis were made by CSF inoculation in healthy guinea-pigs. Definitive cure of CNS and systemic disease were achieved with anti-leptospiral horse serum administration in CSF and blood.	*L. icterohaemorrhaegiae*
Sakellaridis et alI[15] (2009)	59	Greece	Case report	0/1	Not mentioned	Serology	-	Meningoencephalitis. MRI scan showed bilateral lateral ventricular enlargement. CSF with increased signal intensity on T1 and decreased on T2. Pathological FLAIR signal. Patient died of acute myocardial infarction. The patient presented a high protein level in CSF, almost equal than the blood protein level.	-
Waggoner et al[27] (2015)	25 (mean)	Travellers	Case series	0/2	Lymphocitic predominance	CSF PCR	-	The two patients had diagnosis with CSF PCR, targeting de 16S rRNA gene.The second patient	

								had a bacterial load in CSF 5- to 10-fold higher than the plasma. His seric serologies were all negative.	
Middleton[63] (1955)	42	England	Case report	0/1	Lymphocytic pleocytosis (95%)	MAT	2 days	Puppy pet examined with evidence of *L.canicola* infection. The patient developed a neuralgic amyotrophy.	*L. canicola*.
Marotto et al[64] (1997)	4-14	Brazil	Retrospective cohort	-	Lymphocytic pleocytosis more common (54.4±10.7%)	MAT	-	23% of cases with meningitis, without neurologic complications or sequelae.	-
Cargill et al[19] (1947)	-	United States of America	Prospective cohort	12/1	Lymphocytic pleocytosis more common	MAT	-	14 cases of Weil's disease, and 13 with abnormalities in CSF. Only six with clinical signs of meningeal irritation. Xantochromy in 90% of patients (yellow CSF).	-
Dittrich et al[8], (2015)	25 (mean)	Laos	Prospective cohort	-	Mean CSF neutrophils of 30/mm^3 and lymphocytes of 21.8/mm^3.	CSF PCR	-	12% of all patients had evidence of leptospiral infection. 23% patients with meningitis, 45% with meningoencephalitis, 7% with acute encephalitis syndrome, and 26 without fulfilling WHO criteria for both meningitis/encephalitis. 3/23 died.	-

Bhatt et al[65] (2018)	19	India	Case report	0/1	Lymphocytic pleocytosis	CSF PCR	2	MRI revealed multiple hyper intensities in bilateral medial temporal lobes, pons and midbrain suggestive of viral encephalitis. Psychologic sequelae were observed.	-
Puca et al[11] (2016)	18	Albania	Case report	0/1	Lymphocytic pleocytosis (100%)	Serology	<4-5	The patient presented an acute encephalitis as the initial presentation of the disease	-
Bigham[66] (1953)	25	United States	Case report	1/0	Lymphocytic pleocytosis	MAT	12	-	*L. grippotyphosa*
Fox et al[10] (1951)	11	Scotland	Case report	0/1		Complement fixation tests and MAT	<4	-	*L. canicola*
Mondal et al[67] (2014)	32	India	Case report	1/0	Lymphocytic pleocytosis (70%)	Serology	-	Patient presented with a concomitant pancreatitis.	-

*Some patients were eliminated, for presenting mild cell count, not configuring an inflammatory CSF. #Interval between symptom onset and lumbar puncture.

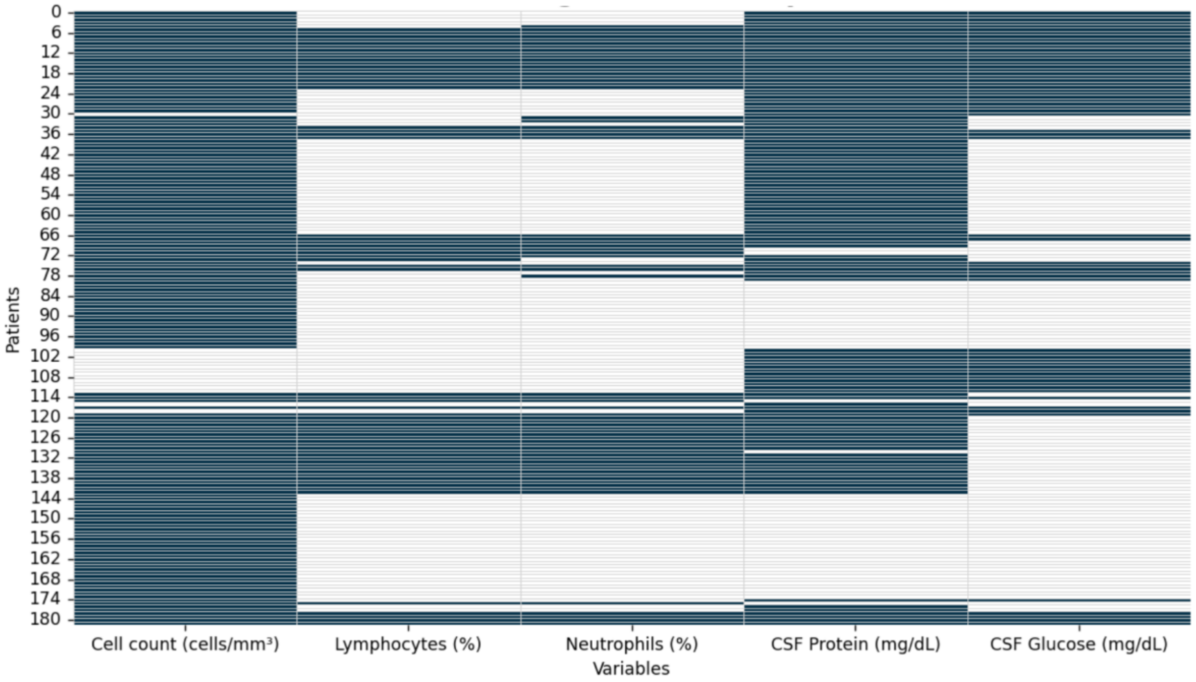

Figure 1. Heatmap summarizing missing data patterns across cerebrospinal fluid (CSF) parameters from literature and the three reported cases. The dataset combines variables extracted from published studies reporting CSF findings in leptospirosis-associated meningitis, together with the three additional cases described in the present work. Studies included: Z-Y.

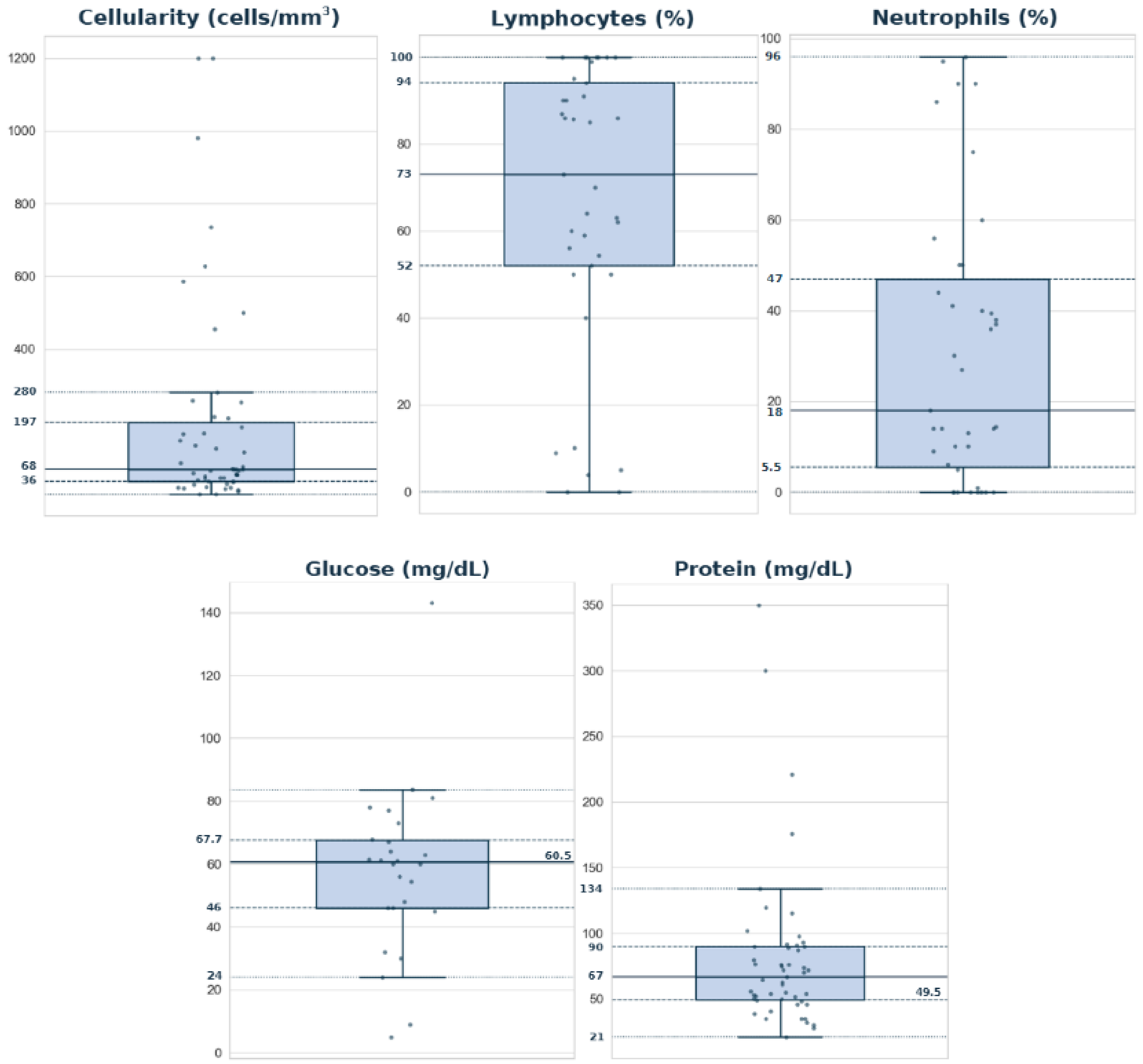

Figure 2. Boxplot of cerebrospinal fluid (CSF) parameters from literature and the three reported cases. Extremely high outlier values that distorted the graphical visualization were removed prior to plotting (protein > 400 mg/dL and glucose > 150 mg/dL). Studies included: Z-Y.
