# Supplementary material for: Leptospirosis-associated meningitis in an urban tropical endemic setting in northeastern Brazil: Three New cases and a meta-summary of 176 reported cases
Source: PLoS Negl Trop Dis. 2026 May 11;20(5):e0014315. doi: 10.1371/journal.pntd.0014315 (PMC13178981; doi:10.1371/journal.pntd.0014315)
Supplement: Attachment — Submitted filename: Response to Reviewers.docx [file pntd.0014315.s002.docx]

Dear Editor-in-Chief

Paul Brindley

co-Editor-in-Chief

Shaden Kamhawi

Section Editor

Elsio Wunder Jr

Dear Academic Editor

Yung-Fu Chang

PLOS Neglected Tropical Diseases

The authors would like to thank the reviewer for carefully evaluating our manuscript and for the constructive comments provided. We have addressed all the reviewers’ questions, as detailed below. All revisions and modifications have been incorporated into the manuscript using track changes and/or highlighted in yellow for ease of review. We believe that these changes have strengthened the quality and clarity of the work. We hope that the revised manuscript is now suitable for publication in the journal.

Best regards,

**Methods**

**Reviewer #1: Methods are OK but clarification/further details should be given to what constitutes lab confirmation of a positive leptospirosis case.**

*Answer: The authors agreed and included a better deviation of a leptospirosis case and laboratorial analysis performed. This criteria was included as a news paragraph as “A leptospirosis case was defined as a patient presenting with clinical features compatible with leptospirosis (e.g., acute febrile illness with headache, myalgia, conjunctival suffusion, or jaundice), with a relevant epidemiological exposure (such as contact with contaminated water or soil, occupational or recreational exposure, or residence in/travel to endemic areas), and laboratory confirmation by serology (IgM ELISA or microscopic agglutination test), PCR, or culture. LAM were defined as a leptospirosis confirmed case of leptospirosis with the presence of compatible meningeal/encephalical syndrome*[*^9^*](https://jidc.org/index.php/journal/article/view/20351461/368)*^,^* [*^22^*](https://pmc.ncbi.nlm.nih.gov/articles/PMC7454101/)*^,^.”*

**Reviewer #2: Well-structured and methodologically thoughtful, especially the integration of clinical cohort and meta-summary. However, why 3 cases chosen must be answered in the background of high volume literature review cases; why not this is a simple case series with review literature rather making cohort + meta-summary? If it can't be justified, better manuscript be revised as simple case series with review literature. Definitions are appropriate; consider streamlining for readability by reducing repetition and consolidating overlapping criteria. The literature review methodology would benefit from greater rigor/clarity (e.g., search timeframe, selection process, potential bias). Justification for outlier exclusion thresholds is good; can be strengthened by briefly citing or referencing expected biological ranges.**

*Answer: The authors agreed that the inclusion of the three new cases not only strengthens the neuroinfection cohort but also contributes additional reports of a rare outcome (i.e., leptospiral-associated meningitis) to both the literature and the present study. Moreover, this inclusion aligns with the primary objective of the metasummary, which is to comprehensively gather and synthesize all available cases of a rare condition. Leptospiral-associated meningitis, as a clinical entity, has been described exclusively in case reports and small cohorts, and to date, no study has systematically consolidated these cases to facilitate a more comprehensive understanding of its clinical spectrum, diagnosis, and management.*

*The methodology has been further refined, including explicit definition of the search timeframe, clarification of the selection process, and expanded discussion of potential sources of bias as suggested. We also improved the methodology by specifying the cohort period and including ethics approval. These limitations are more thoroughly addressed in the Discussion section, given the inherent constraints imposed by the rarity of the condition, which precludes more stringent study selection. In addition, regarding outlier exclusion, values above 1300 cells/mm³ for cellularity and >500 mg/dL for protein were not excluded based on predefined biological reference ranges; we also included this information in the text. Rather, these thresholds were identified as markedly discrepant relative to the distribution observed across the included studies. In the absence of a well-established “expected” cerebrospinal fluid profile for leptospiral-associated meningitis—which, in fact, underscores the rationale for conducting this metasummary—these extreme values were considered inconsistent with the overall dataset and were found to substantially distort the boxplot representation. Their exclusion was therefore performed to preserve the interpretability of central tendency and dispersion measures, and this decision is further discussed in the manuscript. We described that clearly in the corrected article version.*

**Results**

**Reviewer #1: Results are appropriate except for the authors' overinterpretation of the infecting serovar from previous publications. Serology CANNOT identify the infecting serovar (multiple publications have proven this) and therefore the table and text need to be revised to reflect this. Additionally, the way that historical publications stated the Leptospira nomenclature is inaccurate. The species should be italicized and the serovar capitalized.**

*Answer: The authors agree and have removed the serovar data/paragraph from the Results, as well as the column that predominantly listed serovars, since most were identified by serology (MAT) and cannot be reliably confirmed. In addition, when mentioning the species in the text, we italicized as suggested.*

**Reviewer #2: Strong descriptive detail and clinically rich cases; consider improving conciseness by focusing on findings most relevant to LAM. Enhance consistency in data reporting (units, structure across cases) to improve readability and comparability. The literature synthesis is valuable; to improve, emphasize key patterns rather than detailed enumeration (e.g., highlight dominant trends over listing serovars). The CSF analysis is a major strength; consider linking findings more explicitly to clinical implications.**

*Answer: We thank the reviewer for the insightful and constructive comments regarding the clarity, structure, and focus of the manuscript. In response, we have carefully revised the case descriptions to improve conciseness by emphasizing findings most relevant to leptospiral-associated meningitis, while removing less directly contributory clinical and laboratory details. We also standardized the reporting format across all cases, ensuring consistency in units, clinical parameters, and overall structure to enhance readability and comparability. Regarding the literature synthesis, we have revised the text to emphasize predominant patterns rather than extensive itemized descriptions, highlighting the main trends observed across studies, particularly CSF profiles and their variability. Furthermore, we strengthened the discussion of cerebrospinal fluid findings by more explicitly linking CSF patterns to clinical interpretation and diagnostic implications in LAM. We believe these modifications have significantly improved the clarity, clinical focus, and interpretative strength of the manuscript.*

**Conclusions**

**Reviewer #1: Conclusions are OK EXCEPT for the assumption that serology can determine the infecting serovar (see above).**

*Answer: The authors agreed and removed this assumption from the manuscript as suggested.*

**Reviewer #2: The conclusion is missing in main text, however in abstract, it is solid but could be sharpened with a clearer clinical or research-oriented closing statement.**

*Answer: The authors agreed and included a conclusion statement in the final manuscript as well as a clearer clinical and research-oriented closing statement as the following “Leptospiral involvement of the CNS remains an underrecognized and likely underreported cause of aseptic meningitis, as reflected by its low frequency in our cohort despite occurring in an endemic setting. The marked variability in CSF findings underscores the diagnostic complexity of this condition and limits reliance on classical laboratory patterns. Differences in detection rates across studies highlight the importance of sensitive molecular techniques, which may reveal a higher true burden of disease. Current evidence suggests a multifactorial pathogenesis, involving both direct bacterial invasion and immune-mediated mechanisms, with host factors influencing clinical expression and disease persistence. From a clinical perspective, leptospirosis should be considered in the differential diagnosis of aseptic meningitis in endemic areas, particularly in the presence of relevant epidemiological exposures, as early recognition and timely antimicrobial therapy may improve outcomes. From a research standpoint, expanding access to molecular diagnostics and conducting well-designed prospective studies are essential to better characterize disease burden, elucidate underlying mechanisms, and guide evidence-based diagnostic and therapeutic strategies for leptospiral CNS involvement.”*

*”*

**Editorial and Data Presentation Modifications?**

**Reviewer #1: (No Response)**

**Reviewer #2: This is a high-quality, well-researched manuscript with strong novelty in combining clinical data and meta-summary.**
**Key improvements lie in enhancing clarity, reducing density, and emphasizing your unique contribution and clinical relevance. Tightening the narrative will significantly improve readability and publication impact without altering content depth**

*Answer: The authors agreed and decided to tighten the narrative to better improve readability and publication impact as suggested.*

**Summary and General Comments**

**Reviewer #1: This is a thorough review of characteristics of aseptic meningitis due to leptospirosis. I think the authors should emphasize strongly that this is likely underreported as a cause of meningitis (the authors have referenced previous studies that looked at PCR diagnosis). The authors also will need to edit the text and table since serology cannot identify the infecting serovar. This was a historic but inaccurate belief, but multiple publications have cautioned that serology cannot identify the infecting serovar.**

*Answer:The authors agree, and we have included in the Discussion and Conclusion sections aspects related to the underreporting of leptospirosis as a cause of meningitis, as follows: discussion: “Molecular studies in LAM have expanded understanding of both diagnostic strategies and disease pathophysiology*[*^8^*](https://www.thelancet.com/journals/langlo/article/PIIS2214-109X(14)70289-X/fulltext)*^,^*[*^13^*](https://www.frontiersin.org/journals/neurology/articles/10.3389/fneur.2022.1021364/full)*^,^*[*^24^*](https://www.scielo.br/j/mioc/a/mKMjPMhQ4YtSqGng3vh5p4S/?lang=en)*^,^*[*^27^*](https://doi.org/10.4269/ajtmh.15-0174)*^,^*[*^44^*](https://www.nejm.org/doi/10.1056/NEJMoa1401268)*^,^*[*^54^*](https://www.sciencedirect.com/science/article/pii/S0923250803001669?via%3Dihub)*^,^*[*^55^*](https://doi.org/10.1093/infdis/172.1.281)*. Evidence suggests that CSF PCR is more sensitive than serological assays, enabling earlier diagnosis and therapeutic intervention*[*^24^*](https://www.scielo.br/j/mioc/a/mKMjPMhQ4YtSqGng3vh5p4S/?lang=en)*. However, its use remains limited in many endemic regions, contributing to the underrecognition of LAM in patients presenting with aseptic meningitis*[*^15^*](https://doi.org/10.1097/smj.0b013e3181a0ae80)*^,^*[*^24^*](https://www.scielo.br/j/mioc/a/mKMjPMhQ4YtSqGng3vh5p4S/?lang=en)*. Importantly, the detection of Leptospira DNA in patients with LAM does not necessarily indicate active infection, as it may reflect residual genetic material rather than viable organisms capable of replication*[*^15^*](https://doi.org/10.1097/smj.0b013e3181a0ae80)*. These findings suggest that leptospiral aseptic meningitis is likely substantially underreported as a cause of meningitis, particularly in settings where molecular diagnostic tools are not routinely available. Prior studies incorporating PCR-based approaches have demonstrated higher detection rates, reinforcing the notion that the true burden of LAM may be underestimated in the existing literature.” and Conclusion “Leptospiral involvement of the CNS remains an underrecognized and likely underreported cause of aseptic meningitis, as reflected by its low frequency in our cohort despite occurring in an endemic setting.” We have revised the table and the text as suggested, and, since most diagnostic methods were based on serology (MAT), we removed the corresponding table column. We have also included this as a limitation, as follows: “Finally, it was not possible to further characterize the serovars involved in the studies, as most diagnoses were based on serology/MAT, which cannot reliably identify the infecting serovar*[*^55^*](https://doi.org/10.1093/infdis/172.1.281)*.”*

Reviewer #2:

**Few points about ABSTRACT: Strong structure and clear integration of cohort + meta-summary; to improve further, clarify the novel contribution more explicitly (e.g., first combined CSF profile synthesis).**

*Answer: We thank the reviewer for this insightful comment. In response, we have revised the abstract to more explicitly highlight the novel contribution of our study. Specifically, we now clearly state that this work represents, to the best of our knowledge, the first combined synthesis integrating cerebrospinal fluid (CSF) profiles from both an institutional cohort and published cases of leptospiral aseptic meningitis. This clarification has been incorporated into the Conclusion section of the abstract to emphasize the originality and added value of our analysis. We believe this modification strengthens the presentation of the study’s main contribution and improves clarity for the reader.*

**The results are well presented, but could be strengthened by briefly quantifying key pooled findings (e.g., median CSF values) to increase impact.**

*Answer: The authors agreed and included the median CSF values, and the range, in the abstract, to enhance the clinical impact of the manuscript, as follows: “Among 179 patients, including 176 identified in the medical literature, mean CSF values were: cellularity 68 cells/mm³ (range, 30–7800), lymphocytes 73% (0–100), neutrophils 18% (0–96), glucose 60 mg/dL (0–140), and protein 67–90 mg/dL (31–2590).“*

**Conclusion is comprehensive; consider slightly sharpening the clinical takeaway (e.g., when to suspect LAM in practice).**

*Answer: The authors agreed and included the following phrase “From a clinical perspective, LAM should be considered in the differential diagnosis of aseptic meningitis in endemic areas, particularly in the presence of relevant epidemiological exposures and clinical signs of leptospirosis, as early recognition and timely antimicrobial therapy may improve outcomes.” in the conclusion paragraph is a takeaway message to clinical practice.*

**Few remarks on INTRODUCTION: Excellent epidemiological grounding and regional relevance that enhances further by tightening redundancy in pathophysiology description. The knowledge gap is clearly identified; to strengthen it, more explicitly state why prior literature is insufficient (methodological limitations, heterogeneity). The final paragraph is strong but could benefit from a more concise and focused study aim statement.**

*Answer: We thank the reviewer for the positive assessment of the Introduction and for the constructive suggestions. In response, we have revised this section to improve conciseness by reducing redundancy in the pathophysiology description. We have also strengthened the statement of the knowledge gap by more explicitly highlighting the main limitations of prior literature, including small sample sizes, reliance on case reports and small series, heterogeneous diagnostic criteria, and variability in laboratory and CSF methodologies, which collectively limit comparability and synthesis of findings. Finally, we have revised the last paragraph to provide a more concise and focused statement of the study aims, clearly distinguishing the institutional cohort description from the literature-based meta-summary. We believe these modifications have improved the clarity, scientific rigor, and overall focus of the Introduction.*

**Similarly DISCUSSION: Highly comprehensive and intellectually strong, particularly in pathophysiology and literature integration. To improve, enhance focus and prioritization, the discussion is dense and would benefit from clearer highlighting of the most important insights. Strengthen direct linkage to your findings (cohort + meta-summary) rather than extended general literature exposition. Some historical and mechanistic details, while interesting, could be condensed to maintain narrative clarity and impact.**

*Answer: We appreciate this insightful feedback and fully agree with the points raised. In response, we have revised the Discussion to improve focus and prioritization. Specifically, we incorporated additional data from our own cohort and the meta-summary to better complement and contrast the existing literature. Furthermore, we streamlined the section to reduce excessive historical and mechanistic detail, placing greater emphasis on the key clinical findings and the pathophysiological insights directly supported by our cohort and meta-summary.*

**The limitations section is appropriate; consider making it slightly more explicit and structured**

*Answer: The authors agree and made the limitations paragraph more explicit and structured as the following “This study has limitations that should be acknowledged.This study has several limitations. First, the diagnosis of LAM was not confirmed by direct detection of Leptospira in CSF, which may limit diagnostic certainty. However, all cases met accepted diagnostic criteria based on compatible clinical and epidemiological features in conjunction with positive serology, supporting the validity of the diagnoses. Second, the available literature on LAM is scarce and frequently characterized by incomplete reporting and substantial heterogeneity in clinical, laboratory, and diagnostic data, which constrains comparability and synthesis. Third, the retrospective design of this study may be subject to information bias and limits the ability to establish temporal and causal relationships. Finally, it was not possible to further characterize the serovars involved in the studies, as most diagnoses were based on serology/MAT, which cannot reliably identify the infecting serovar*[*^55^*](https://doi.org/10.1093/infdis/172.1.281)*.”*

*In addition, as part of the manuscript revision, we have also improved overall readability and conciseness, as well as English grammar and consistency of abbreviations.*
